# Life Cycle Assessment of Advanced Circulating Fluidized Bed Municipal Solid Waste Incineration System from an Environmental and Exergetic Perspective

**DOI:** 10.3390/ijerph181910432

**Published:** 2021-10-03

**Authors:** Jun Li, Lixian Wang, Yong Chi, Zhaozhi Zhou, Yuanjun Tang, Hui Zhang

**Affiliations:** 1 State Key Laboratory of Clean Energy Utilization, Zhejiang University, Hangzhou 310027, China; 21860062@zju.edu.cn (J.L.); wanglixian@zju.edu.cn (L.W.); zhanghui626@zju.edu.cn (H.Z.); 2 Zhejiang Development & Planning Institute, Hangzhou 310012, China; 11527033@zju.edu.cn; 3 Department of Energy and Environment System Engineering, Zhejiang University of Science and Technology, Hangzhou 310023, China; tang@zust.edu.cn

**Keywords:** environmental life cycle assessment, exergetic life cycle assessment, municipal solid waste, mechanical biological treatment, environmental burdens, resource use efficiencies

## Abstract

The production of clean and efficient energy from municipal solid waste (MSW) is extremely urgent matter due to an increasing energy demand and environmental concerns. In this study, a high steam parameter (520 °C, 7.9 MPa) circulating fluidized bed (CFB) MSW incineration system, equipped with a mechanical, biological treatment and external heat exchanger systems, was introduced and a comparative study with a typical mechanical grate (450 °C, 5.3 MPa) incineration system and conventional CFB (485 °C, 5.3 MPa) incineration system was carried out from a life-cycle, environmental and exergetic perspective which could assess different energy and material outputs based on real operating data. Moreover, the potential system optimization of this advanced CFB system was proposed. The results showed that the advanced CFB system was more environmentally friendly and resource-efficient than conventional MSW incineration systems. The recovery of material should be given priority over energy recovery. According to the assessment of the environment, and energy and material recovery, a process improvement with an incinerated refuse-derived fuel and a semi-compost produced by MBT as a soil conditioner was highly recommended.

## 1. Introduction

The generation of municipal solid waste (MSW) has increased dramatically with the vast growth of population and urbanization in recent years in China. According to the official reports [1], in China there were 158.05 and 215.21 million tons of MSW produced in 2010 and 2017, respectively, corresponding to an annual increase rate of 4.5%. Meanwhile, the relevant harmless treatments for MSW were also developed, especially the MSW incineration technology due to its advantages for significant waste mass and volume reduction, complete disinfection, and energy recovery [2]. However, the electricity recovery efficiency of the MSW incineration plant is usually as low as approximately 20%. The reasons for this are mainly attributed to not only the low-grade fuels, but also to the low steam parameters due to the high-temperature HCl corrosive effect [3]. The chlorine-containing MSW brings chlorine to flue gas which leads to the emergence of HCl [4]. At a temperature above 450 °C, acid flue gas containing SO_2_ and HCl can corrode the heat transfer surface [5].

To obtain a higher energy recovery from MSW, an advanced circulating fluidized bed (CFB) incineration system equipped with a mechanical biological treatment (MBT) system and final steam superheater used as external heat exchanger (EHE) located in solids return, was investigated and constructed to achieve high steam parameters in order to make the MSW incineration system more environmentally friendly and energy efficient, while its environmental savings, resource benefits, and energy efficiencies lacked a quantitative evaluation.

For part of the MBT system, the integrated material recovery and valorization system improve resource efficiency. The poly-generation of the MBT system enhances product diversification and minimizes financial risks [6]. MBT has an optimal net environmental impact for MSW treatment [7]. In short, due to material recovery and fuel upgrading, the MBT system is considered a promising method for MSW treatment [8]. A more detailed description concerning the equipment is clarified in the Supplementary Materials.

**Figure 1:**
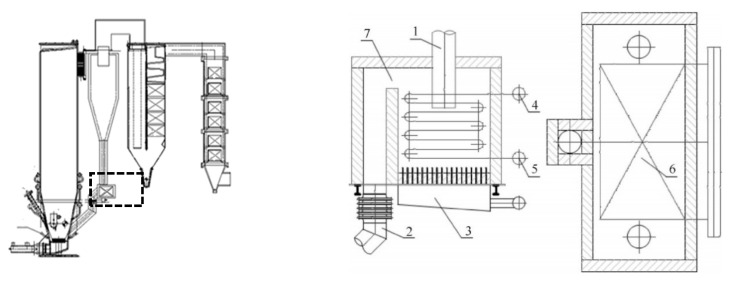
The figuration of EHE: 1—ash inlet 2—ash back 3—equalizing air chamber 4—high temperature superheater outlet header 5—high temperature superheater inlet header 6—high temperature superheater serpentine tube 7—ash back overflow hole.

For the part of the EHE system, an external heat exchanger (EHE) located in the solids returns is widely used in large CFB systems, as shown in Figure 1 [9]. The EHE is beneficial for making the boiler load adaptable and for regulating the bed temperature [10]. The EHE is convenient and flexible for the design of heating surfaces, owing to its adjustable solid flow rate and good heat transfer characteristics [11]. The EHE works as a high temperature heat transfer that exchanges heat between the flowing steam inside the transfer and solid particles instead of flue gas. Therefore, it helps obtain a high steam parameter and high electricity recovery, especially in MSW incineration plants without the issue of the high-temperature HCl corrosive effect which strictly limits the main steam parameters (usually below 450 °C, 4.0 MPa). However, in our study, with the use of EHE, the main steam parameters can reach up to 520 °C and 7.9 MPa and help achieve energy recovery efficiency as high as 28%. The EHE system uses high-temperature ash and waste particles as a heat resource where there is no participation of corrosive flue gas. This is why this system does not present the problem of the HCl corrosive effect.

The life cycle assessment (LCA) is a holistic evaluation methodology that quantifies all the environmental burdens of a product or service from the cradle to the grave throughout the life cycle [12]. The LCA has been widely applied in integrated waste management systems in recent years [13,14,15]. Dong [16] adopted the life cycle assessment to estimate the impacts of different flue gas treatment systems typically found in modern MSW incinerators. Hong [17] conducted a life cycle comparison of environmental performances from four available MSW treatment alternatives (CFB system, MG system, landfill and emitted gases used for power generation and landfill), indicating that CFB system behaved better than MG system, while landfill was the worst environmental option. The modified LCA integrated with an engineering economy for the design experiments [18] was associated with energy consumption, as well as life cycle cost [19], and energy, economy and society [20] was also conducted in the literature. However, the life cycle assessment of the advanced CFB incineration system equipped with the MBT and EHE is not yet studied and needs a comprehensive analysis, based on the on-site operational data.

The LCA is able to identify and quantify energy, material and waste emissions; however, as it is based on the first law of thermodynamics, it encounters many difficulties in application, especially when comparing different energy forms because it considers only quantity but not quality [21,22]. Exergy not only reflects both the quantity and quality of energy carriers by the first and second law of thermodynamics, but represents the value of natural resources too, by means of the unified evaluation standard. When integrated with the LCA framework, the exergetic life cycle assessment (ELCA) is proposed and measures the total exergy of natural resources extracted from the environment in a life cycle of a product or process [23,24]. Szargut [25] summarized the methods of calculating the cumulative exergy consumption (CExC) and introduced the cumulative degree of perfection (CDP) for the entire chain of production processes. The studies on cumulative exergy consumption indicators applied to the Ecoinvent database were conducted with the aim of convenient calculations [26]. Based on the concept of CExC, ELCA was applied to assess “CO_2_ zero-emission” energy systems [22], different sludge treatment techniques [27], and MSW gasification-based thermal treatments [21]. However, the advanced CFB incineration system equipped with MBT and EHE has not been assessed yet. Therefore, the ELCA should also be conducted. Recent studies recognized ELCA as a part of LCA representing the impact category of resources depletion and took environmental burdens into account via the abatement exergy of released pollutants [19,21], while the land resources were not considered.

The resource value of land can be defined as the total natural potential net primary production (NPP) of a land area if it is not occupied by humans [28,29]. Due to the depletion of non-renewable resources and policy actions to mitigate climate change, an increased pressure on land as a resource is to be expected [30,31]. The ELCA is suggested to bring both abatement exergy and land use into consideration in its framework for a comprehensive and scientific evaluation.

The goal of this study is to compare the environmental impact, material and energy efficiency of three waste-to-energy cases by LCA and ELCA, in order to advise waste treatment. This study evaluated the environmental burdens and resource use efficiencies of the advanced CFB incineration system equipped with MBT and the EHE. The conventional CFB and MG incineration systems were compared with this advanced CFB system using the methods of environmental LCA and exergetic LCA. The two assumed process improvements, corresponding to whether semi-compost should be recycled as soil conditioner or sent to landfill sites, were modeled and compared with the current operation of this advanced CFB system. All the data used for the calculation were collected from real operational report and records, and the functional unit was unified as 1000 kg of raw MSW, and the plants selected were representative in China. The LCA was used to measure environmental burdens, while the ELCA quantified the relevant resource use efficiencies. Afterward, the considered ELCA results with land use were compared with the case of not taking land use into account. The final results could serve as the scientific basis for further developing advanced MSW incineration systems in the future.

## 2. Methodology

As guided by the International Organization for Standardization (standards 14040 series) [32], LCA consisted of four steps: goal and scope definition, life cycle inventory (LCI), life cycle impact assessment (LCIA) and interpretation. When collecting life cycle inventories, the environmentally relevant physical flows to and from the systems should be included. The LCA method, Recipe 2016, was used for environmental assessment in this study. Due to a more specific impact category of midpoint compared to endpoint, midpoint was chosen and analyzed. Six environmental impacts were considered: climate change, fossil depletion, human toxicity, ironizing radiation, particulate formation, and photochemical oxidant formation, because their midpoint impacts contributed more than 98% of the total impact while the influence of the remaining 11 indicators was too small to analyze. The negative value represented gains to the environment. The characterization factors were adopted from GaBi 8.0 software.

ELCA was proposed and developed by combining the concept of exergy analysis and environmental LCA. Consequently, the general framework of ELCA was recognized as identical compared to LCA [21]. The life cycle-based exergy analysis quantified the cumulative exergy consumption of a system from cradle to grave. Thus, ELCA could be supplemented as part of LCA [33], representing one impact category to assess resources consumption. Szargut [25] proposed cumulative exergy consumption (CExC) to measure ELCA [34]. CExC represented the total exergy of natural resources delivered to the system in all links of the production chain that started with resource exploitation and finally led to the product [25,34].

In this study, the calculation of CExC was perfected and land use was taken into consideration. The natural potential net primary production (NPP), which was the amount of NPP a land area would produce if it was not occupied by humans [28,29], could be used as a suitable proxy to represent the resource value of the land. Alvarenga [35] proposed and implemented a new framework to calculate exergy-based spatial explicit characterization factors (CF) for land as resources. The CExC of a system was calculated by summing all the CExC of life cycle inventories, while the CExC of certain LCI could be obtained from additive exergy of different natural resources, including land resources. The CExC efficiency (η_CExC_) was defined as “the ratio of all CExC of the output products and input resources” and calculated as shown in Equation (1) [19]:

(1) $$\eta_{CExC}=\frac{{\left(\sum_{i}O_{i}\right)}_{CExC}}{{\left(\sum_{j}I_{j}\right)}_{CExC}}=\frac{{\left(O_{useful}\right)}_{CExC}}{{\left(I_{MSW}+I_{electricity}+I_{hydrated\_\lim e}+I_{active\_carbon}+I_{ammonia}+I_{diesel}\right)}_{CExC}}$$

where, “*I*” represented the input flows, including MSW, electricity, hydrated lime, active carbon, titanium dioxide, ammonia, cement, diesel oil, and coal; “*O*” referred to the output flows, which were different useful products, mainly electricity.

Based on LCI results and CExC method, cumulative degree of perfection (CDP) could be used to evaluate the degree of thermodynamic imperfections of the selected thermal conversion systems. CDP was proposed and defined as the ratio of exergy content of the products to the total CExC of the input energy and materials as presented in Equation (2) [36]:

(2) $$CDP=\frac{e_{X,useful}}{{\left(I_{MSW}+I_{electricity}+I_{hydrated\_\lim e}+I_{active\_carbon}+I_{ammonia}+I_{diesel}\right)}_{CExC}}$$

where, “*e*_*X,useful*_” represented the exergy of the useful products.

In addition, ELCA could also be applied to assess the environmental impacts by taking into account the exergy consumption for the treatment of emissions to the environment [37]. Abatement exergy (AbatEx) was thus defined as “the internal exergy loss caused by the abatement of air emissions to an accepted limit for environment” [38]. Cornelissen [36] calculated the abatement exergy of CO_2_ at 5.86 MJ/kg, which meant the exergy required to store 1 kg of CO_2_ in a depleted oil well at 8 MPa was 5.86 MJ. The abatement exergy of SO_2_ and NO_x_ was 57.0 and 16.0 MJ/kg, respectively [36]. The development of the abatement exergy calculated for air emissions was limited due to the lack of available data in the literature [38]. Therefore, the abatement exergy was subtracted from the output CExC flows, and the indicator of AbatCExC efficiency (η_AbatCExC_) is proposed in Equation (3) [36]:

(3) $$\eta_{AbatCExC}=\frac{{\left(\sum_{i}O_{i}\right)}_{CExC}-AbatEx}{{\left(\sum_{j}I_{j}\right)}_{CExC}}=\frac{{\left(O_{useful}\right)}_{CExC}-AbatEx}{{\left(I_{MSW}+I_{electricity}+I_{hydrated\_\lim e}+I_{active\_carbon}+I_{ammonia}+I_{diesel}\right)}_{CExC}}$$

where “AbatEx” represented the abatement exergy required for treating air emissions, mainly CO_2_, SO_2_ and NO_x_.

### 2.1. System Boundary

**Figure 2:**
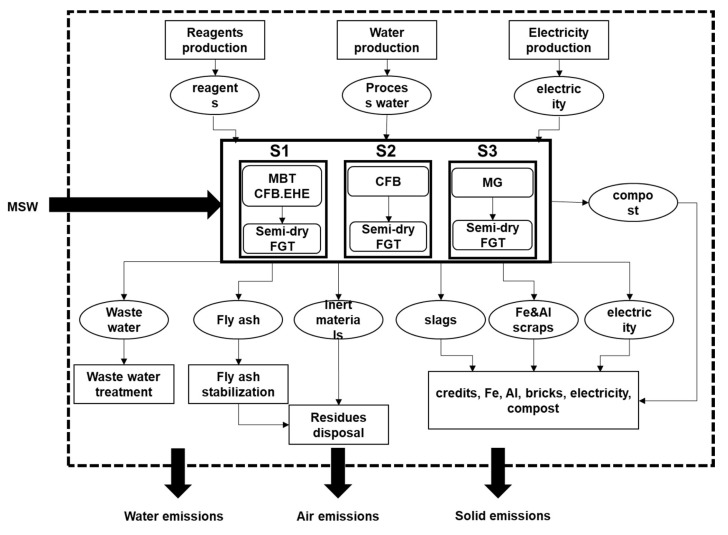
System boundary and flow charts of the considered incineration systems.

Figure 2 provided an overview of the system boundary and flow charts of the three typical MSW incineration systems. The evaluation started with the collection of MSW into the incineration plant and ended with the emissions of the plant to air, water and soil. The consumed reagents, water and electricity were traced back to their production processes; therefore, the indirect environmental impacts or resource use efficiency could be obtained. The produced waste water was treated inside the plant and fly ash was landfilled after stabilization. The inert materials sorted from raw waste were landfilled as well. Useful resources like ferrous and aluminum metals were recycled. Furnace slags were considered as valuable resource for substituting bricks and the electricity was recovered to electricity grid of China. Optionally, the produced semi-compost could be used as soil conditioner substituting a certain amount of phosphate fertilizer.

In this study, five MSW incineration scenarios were considered. The advanced CFB incineration system equipped with MBT and the EHE system was considered as the base system (S1). For this advanced CFB system, two general downstream semi-compost utilities were investigated by using the semi-compost as soil conditioner (S1_soil conditioner_) or by landfilling the semi-compost (S1_landfill_). S2 represented the conventional CFB incineration system and S3 was the current, prevailing MG incineration system. The specific descriptions of the five scenarios can be found in the part “Specific descriptions of the five scenarios”, Figure S1 and Figure S2 of the online Supplementary Materials. Based on the proposed schemes, two parallel comparisons (S1 vs. S2 vs. S3, and S1 vs. S1_soil conditioner_ vs. S1_landfill_, respectively) were conducted from the environmental burdens and exergetic resource use efficiencies using LCA and ELCA.

### 2.2. Description of the Proposed Scenarios

The qualitative analysis of life cycle inventory data determined directly the effectiveness of an LCA or ELCA. In this study, data used for calculation were mainly from on-site operational records, except for the parts of ELCA and the potential optimization assessment. The typical values for characterization of MSW, which were used for the considered scenarios, are specified in Table 1. Zhou [39] reviewed the characteristics of physical and chemical composition of MSW in China; the mean value was referred to in this study as shown in Table 1. It appeared that the MSW of 5 scenarios was similar to the Chinese average level.

#### 2.2.1. S1: Advanced Circulating Fluidized Bed Incineration System in Zibo

**Figure 3:**
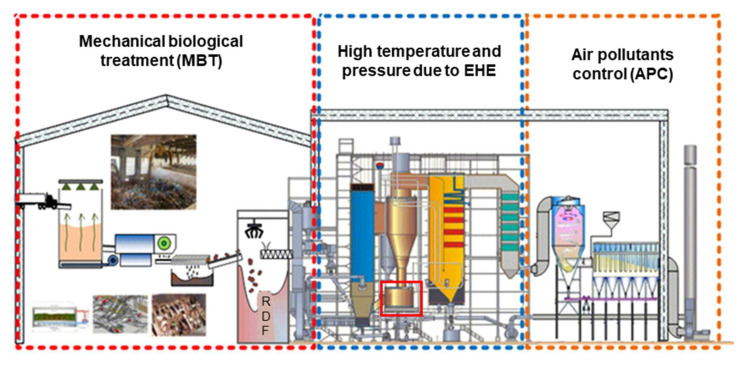
The technological process of advanced CFB incineration system.

S1 was the advanced type of circulating fluidized bed in China, supported by a complex mechanical biological treatment system for treating raw waste before combustion, was designed to reach a relative high steam parameters level (520 °C, 79 bar). Mechanical biological treatment (MBT) of mixed streams is becoming increasingly popular as a method for treating MSW [40]. The outputs of MBT plants were: recyclable (mostly metals) and compostable materials, refuse-derived fuel (RDF) and a fraction of residuals. Differently, the selected plant, S1, took MBT as a part to treat raw waste, the advantage was that after biological reaction and mechanical screening, RDF with low water content and high calorific value could be obtained, together with useful resources, such as ferrous and non-ferrous (aluminum) metals, as well as semi-compost burnt in incinerator. MBT is aerobic and more details on MBT can be found in the Supplementary Materials. The reason why S1 could reach relatively high steam parameters was that the final steam superheater took place in the heat exchangers located in the solids returns, as shown in Figure 3, so that they prevented the contact between high-temperature flue gas and heat transfer. As a result, the configuration not only guaranteed a high temperature and pressure steam but also reduced the fouling and corrosion of the super heat transfer surfaces. As for the air pollution control system, S1 was composed of selective non-catalytic reduction technology, semidry-dry scrubber, active carbon absorption, as well as bag house filter.

#### 2.2.2. S1_soil conditioner_: Advanced Circulating Fluidized Bed Incineration System with Semi-compost Used as Soil Conditioner in Zibo

Generally, one potential advantage of MBT technology over the waste incineration plant was the production of semi-compost that could be used as phosphate fertilizer or soil conditioner, which was good for soil and planting. Thus, S1_soil conditioner_ with the utilization of semi-compost sorted from MBT as soil conditioner for farmland, was also proposed and evaluated in this study, even though its commercial application is still under research. The APC system and the treatment of solid residues of S1_soil conditioner_ were almost the same as S1.

#### 2.2.3. S1_landfill_: Advanced Circulating Fluidized Bed Incineration System with Landfill Disposal of Semi-Compost in Zibo

S1_landfill_ was represented as a variant of the advanced CFB system, which was set to send the produced semi-compost to landfill site in Zibo. The landfill disposal of semi-compost was chosen because, on the one hand, semi-compost sorted from the MBT system had a high content of ash which could cause adverse effects to the combustion; on the other hand, semi-compost could be used for fertilizer/soil conditioner, which was not previously studied in depth. Compared to S1 and S1_soil conditioner_, the same APC system and solid residues treatment methods were adopted in S1_landfill_.

#### 2.2.4. S2: Conventional Circulating Fluidized Bed Incineration System in Hangzhou

S2 represented the conventional circulating fluidized bed MSW incineration system in Hangzhou, China, with 485 °C and 53 bar boiler parameters. Available from 2015, this 24 MW system handles approximately 400,000 tons of MSW per year. Though S2 also had an external heat exchanger to produce superheat steam, the produced steam was much lower than S1 (485 °C, 53 bar for S2 comparing to 520 °C, 79 bar for S1), and S2 had no pretreatment for raw waste. In this study S2 were regarded as conventional CFB systems. In order to control the emission of flue gas pollutants, the selective non-catalytic reduction (SNCR) equipment and selective catalytic reduction (SCR) system were installed; flue gas treatment facilities composed of a semidry scrubber, active carbon absorption and bag house filters were also used [20].

#### 2.2.5. S3: Moving Grate Incineration System in Zhuji

S3 reflected the MG incineration system in Zhuji, China, which was built to substitute old CFB incineration plant for the improvement of technology and environment protection. The plant based on mechanical moving grate (MG) for the production of electricity (24 MW), and S3 was just a half of completion of the whole construction. The selected MG system, with an annual capacity of 147,782 t, was assumed to be in operation for 30 years as designed. A main advantage of the MG incinerator was its capacity to the treat unsorted waste [41]. Heat recovery steam generated superheated steam (450 °C, 53 bar) with the utilization of heat from flue gas, and steam forced the turbine generator to produce electricity. Regarding air pollution control (APC) systems, S3 was equipped with semi-dry flue gas treatment system (SNCR-SCR, semidry-dry scrubber, active carbon absorption, and bag house filter).

### 2.3. Life Cycle Inventory Analysis

The qualitative life cycle inventories used for calculating LCA and ELCA results were similar, mainly due to the on-site operational records, supplemented by the literature and standard regulations. As for the semi-compost sorted from the MBT system shown in Supplementary Table S1, the Chinese national regulation standard shown in Supplementary Table S2 was used to estimate its feasibility of soil conditioner. According to testing results, the As, Cd, Pb, Cr and Hg content of the semi-compost reached 0.003%, 0.0006%, 0.003%, 0.007%, and 0, respectively. Thus, the regulation was satisfied and the semi-compost could be used as soil conditioner substituting phosphate fertilizer for compost, in the case of this study. Considered in the calculations as the category “offset”, 32.5 kg of compost could replace 1 kg of commercial phosphate fertilizer.

Based on the collected data and assumptions, the inventory data was summarized in Supplementary Table S3, the input and output materials/energy flows were included, and the functional unit was set as one ton of raw MSW. In the appliance with the attributional LCA approach, a mix of energy sources in China were used (63.0% fossil fuel, 16.9% hydropower, 10.4% wind power, 7.2% solar power and 2.5% nuclear power). The fossil fuel mix used here was the combination of 70%+ coal, 10%+ oil and 10%+ natural gas in Chinese power plants.

It was obvious that the emission of NO_x_ in the conventional CFB system (S2) and MG system (S3) was slightly lower than the advanced CFB systems (S1, S1_soil conditioner_ and S1_landfill_). The reason for this was mainly due to the use of SCR technology in S2 and S3.

The background data of ECLA are listed in Supplementary Table S4, and the CExC of materials/energy and the abatement exergy of CO_2_, SO_2_, NO_x_ were included. The NPP of materials/energy were calculated according to the area of land use and the relevant characterization factor (CF) of the land resources. According to the study of Alvarenga [35], the average CFs were 16.0, 26.5, 19.8 and 18.7 MJ/m^2^.year for China, Germany, the USA and Europe, respectively. The chemical exergy of MSW could be calculated by Equations (4) and (5) [42]:

(4) $$e=\beta \times LHV_{MSW}$$

(5) $$\beta =\frac{1.0412+0.216H/C-0.2499O/C\times (1+0.7884H/C)+0.045N/C}{1-0.3034O/C}(O/C\leq 2.67)$$

## 3. Results and Discussion

### 3.1. Environmental Life Cycle Assessment

**Figure 4:**
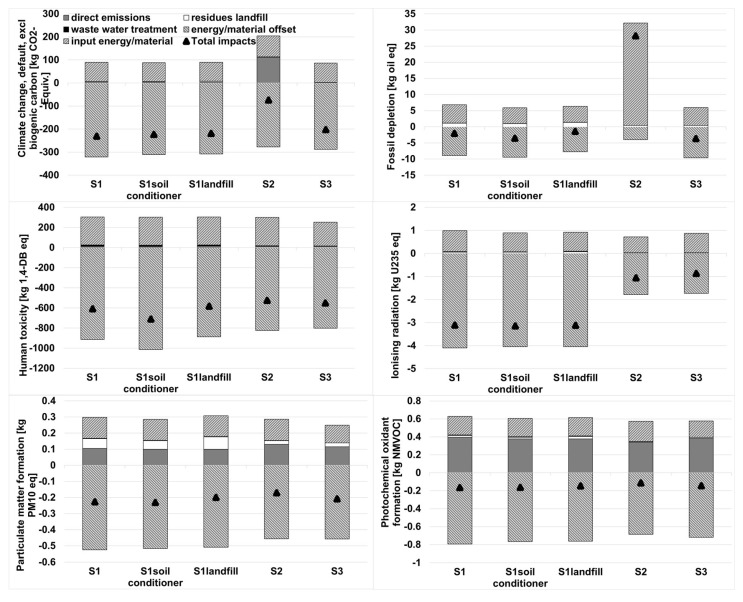
Characterized environmental impacts of the considered systems.

In order to identify the key substances and corresponding stages, the six most important characterized environmental impacts were studied here, including climate change (CC), fossil depletion (FD), human toxicity (HT), ironizing radiation (IR), particulate formation (PF), and photochemical oxidant formation (POF) (Figure 4). The results showed that S1 exhibited lower potential effects than S3, except for the impact of FD, while the potential effects were completely better than the S2 system. As presented in Figure 4, the impact category of FD caused by S1 reached the value of −2.07 kg oil equivalent, higher than the S3 system (−3.62 kg oil-equivalent). The reason for this was mainly attributed to the additional consumption of electricity and diesel for the MBT system pre-treating raw MSW. As mentioned in the Supplementary Materials, the internal electricity consumption rate of S1 increased to 24.9%, while approximately two thirds of the internal power consumption was used to operate the MBT system. Additionally, S3 produced many furnace slags (200 kg for S3 compared to 81.7 kg for S1), which could be used as a substitution for bricks. The material recovery of S3 avoided lots of indirect environmental damages caused by high-pollution industry, such as bricks production. As a result, the environmental saving from slags and the produced electricity of S3 was proven to be higher than the savings from the energy/materials recovered of S1. The impact category of FD caused by S2 increased to 28.2 kg oil equivalent as a positive value, indicating the obvious damage to the environment. According to the life cycle inventory data from Supplementary Table S3, it was obvious that S2 consumed large quantities of coal and diesel to complete the ignition and starting up procedure of the circulating fluidized bed boiler. The higher the amount of electricity recovered and recycled resources were, the higher the gains in environmental protection. The advanced CFB system (S1) performed better than S3 in all cases, mainly due to the higher electricity generation efficiency (28% for S1 comparing to approximately 20% for S3). The recycled ferrous and non-ferrous metals from the MBT system were seen as credits which helped S1 achieve a better environmental score.

The conventional CFB system (S2) consumed more electricity, coal and diesel than the MG system (S3) (89.8 kWh-electricity, 41.8 kg-coal and 2.06 kg-diesel for S2 comparing to 76 kWh-electricity, 0 kg-coal and 0.25 kg-diesel for S3), which increased not only the indirect environmental damages from the relevant production of energy/materials but also the direct emissions, such as fossil CO_2_. Thus, S2 exhibited the highest impact for almost every category.

Among the five different life cycle stages of the impact contributors, the impacts caused by waste water treatment were relatively small, accounting for less than 1.2% of the total environmental burdens, except for the offset from the energy/material recovered. The results were based on the reality that the consumption of reagents for cleaning waste water in power plants was not available. In the waste water treatment stage, the waste water from the operational plants and the leachates from the solid residues landfill were first treated inside the plant and then sent to the waste water treatment plant for deep-cleaning; thus, if the reagents used for treating water were not taken into consideration and only the limited water emissions (like COD, BOD and NH_3_-N) were considered, the environmental impacts generally appeared as a very small value, proportional to the amount of waste water treated. In general, the impacts from the waste water treatment of S1 exhibited higher values than S2 and S3, and the drawbacks mainly resulted from a higher production of waste water from complex pretreating systems and leachates from both biological reactions and residues landfill.

The stage of direct emissions played an import role in PF and POF due to pollutants such as SO_2_, NO_x_, and related particles. In the S2 scenario, the stack CC emission was the highest; subsequently, CO_2_ was found to be the most crucial contributor, as shown in Supplementary Table S3. CO_2_ in the flue gas was a product from the consumption of coal and diesel for combustion or diesel for transportation. However, the CC burdens of S2 could be negated by the environmental credits from the recovery of energy/materials leading to a net environmental saving.

Overall, the environmental improvement of the advanced CFB system (S1) verified a significant role of the external heat exchanger (EHE) located in solid returns, as well as the MBT system, which could not only recycle useful resources like ferrous and non-ferrous metals, but also allowed a higher steam parameter to increase the overall energy efficiency. Additionally, the debugging and stable operation of MBT in an advanced CFB incineration system were of vital importance to its entire environmental performance.

A parallel comparison between the three advanced CFB scenarios (S1, S1_soil conditioner_ and S1_landfill_) was also conducted. It appeared that the incineration plant under the condition of S1_soil conditioner_ performed the best when compared to S1 and S1_landfill_, except for the impact category of CC. As presented in Figure 4, the impact category of CC caused by S1_soil conditioner_ reached the value of −223 kg CO_2_ equivalent and was found to be slightly higher than S1 system (−231 kg CO_2_ equivalent). The environmental savings of using semi-compost as a soil conditioner could not negate the electricity credits by using semi-compost as a fuel, as the resulting S1_soil conditioner_ achieved a lower score for the impact of CC than S1. The semi-compost sorted from the MBT system appeared to have a high ash content and a low calorific value. During the combustion in the incineration, a lot of fly ash was produced and the consumption of relevant reagents increased so that it was not be suitable for combustion to generate power. S1_soil conditioner_ recycled the semi-compost as a soil conditioner which could be seen as a certain amount of phosphate fertilizer, preventing the large environmental damages from producing phosphate fertilizer, as well as reducing the use of the chelating agent for fixing fly ash. So the environmental savings (FD, HT, IR, PF and POF) proved higher than the credits from the electricity produced by the semi-compost in S1. By contrast, S1_landfill_ exhibited the highest impacts for all categories (CC, FD, HT, IR, PF and POF). S1_landfill_ lacked the advantage of environmental credits via the electricity recovered as compared to S1 (371 kWh per t-MSW for S1_landfill_ comparing to 381 kWh per t-MSW for S1). Additionally, the S1_landfill_ semi-compost was seen as containing useless residues and was sent to the landfill sit; therefore, the total amount of residues for landfill increased and more energy/materials (like HDPE and clay) were consumed for this purpose, resulting in an increase in the indirect environmental burdens, as well as the direct emissions, i.e., NH_3_ and CH_4_.

**Figure 5:**
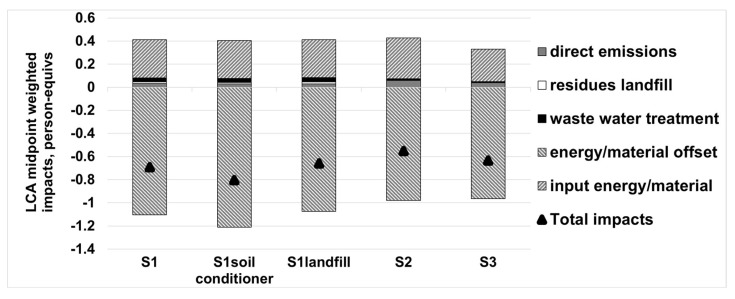
Normalized environmental impacts of the considered scenarios.

As for the whole environmental performance, Figure 5 illustrates the normalized environmental impacts of different scenarios from five different life cycle stages: direct emissions, residues landfill, waste water treatment, energy/material offset, and input energy/material. From Figure 5, the results revealed that the advanced CFB incineration system (S1) exhibited lower environmental burdens as compared to the conventional CFB (S2) and MG (S3) systems. All of the three systems appeared to have negative values, which actually indicated net environmental savings. The most important two stages contributing to the net environmental impacts of the systems, in the order of contribution, were energy/materials offset and input energy/materials. Meanwhile, the total of the remaining impacts came from direct emissions, residues landfill and waste water treatment and only accounted for 17~25.4% of the impacts from input energy/materials, or 4.9~7.8% of the impacts from energy/materials offset.

The impact from the energy/material offset of S1 reached −1.104 person equivalent, and was found to be more significant than S2 (−0.979 person equivalent) and S3 (−0.964 person equivalent). The reason for this was mainly attributed to the use of the MBT pre-treatment system and the EHE in the solids returns; the former recycled useful resources like ferrous and non-ferrous (aluminum) metals. The latter allowed the boiler to employ higher steam parameters than those of a conventional waste boiler (520 °C, 79 bar in comparison to 400 °C, 40 bar in conventional MSW incineration plant [43] so that the electricity generation efficiency improved effectively (electricity production efficiency of 28%). As for the impact from the input energy/materials, the value of S1 was lower than S2 by approximately 0.02 person equivalent, but higher than S3 by about 0.05 person equivalent, which was negligible when compared to the difference of impact from the energy/materials offset. When comparing S2 with S3, and S3 with a lower impact from direct emissions and input, the energy/materials exhibited higher net environmental savings than S2, which lead to the most inferior to normalized environmental performance. Among the advanced scenarios (S1, S1_soil conditioner_ and S1_landfill_), S1_soil conditioner_ exhibited the best normalized environmental performance due to the use of a semi-compost from the MBT system as soil conditioner, substituting phosphate fertilizer. This finding, that the recovery of materials improves the environmental performance of an MSW management system, is also supported by other authors [44,45,46,47].

The conclusion from the characterization of the environmental impacts and normalized environmental impacts showed that the environmental performance of the advanced CFB scenarios varied in a descending order: S1_soil conditioner_ > S1 > S1_landfill_ (“>” means the former system is superior to the latter one). The results clearly indicated the advantages of using semi-compost as a soil conditioner rather than fuels. Therefore, it was recommended that recycle or reuse should always be considered superior to disposal or landfill.

Moreover, the different sensitivity factors of the inputs/outputs were calculated for S1. The sensitivity factor was the ratio of the result change to the input/output variable change. The net electricity sensitivity factor for the normalized results, CC, FD, HT, IR, and POF, were −1.11, −1.17, −0.98, −0.84, −0.89, respectively, meaning the power generation efficiency had a great influence on the environment. Therefore, the efficiency improvement was very necessary. The materials were recycled from MBT (metal, brick and fertilizer) and the sensitivity factors were −0.03, −0.01 and −0.04, respectively. This indicated that fertilizer was the most environmentally friendly output among the recyclable materials. Again, the S1_soil conditioner_ produced the highest net electricity power and recycled compost, and so it had the best performance.

### 3.2. Exergetic Life Cycle Assessment

**Figure 6:**
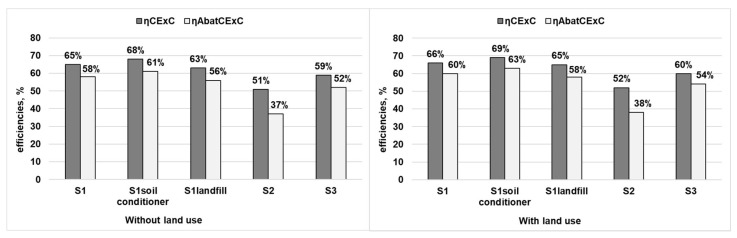
Cumulative exergy consumption (CExC) efficiency and AbatCExC efficiency of the considered systems.

Figure 6 presents the cumulative exergy consumption (CExC) efficiency and AbatCExC efficiency of different scenarios. The CExC efficiency (η_CExC_) of the considered scenarios in descending order were as follows: 65% of S1 > 59% of S3 > 51% of S2, and 68% of S1_soil conditioner_ > 65% of S1 > 63% of S1_landfill_, respectively. If the cumulative exergy consumption of land resources was considered, the ranking results were maintained but the values of each scenario increased; these values were 66%, 69%, 65%, 52%, and 60% for S1, S1_soil conditioner_, S1_landfill_, S2, and S3, respectively. The increments of η_CExC_ seemed reasonable because the main contributor of the difference was the amount of electricity, while all five scenarios exhibited a net recovery of electricity. The input cumulative exergy consumption of electricity increased by less than 45 MJ per t-MSW if the CExC of land resources was taken into consideration, while the output cumulative exergy consumption of electricity increased by more than 160 MJ per t-MSW, approximately four times the input value. Considering that the values of η_CExC_ from the five scenarios were higher than the relevant performances without considering land use, this reflected that the framework which brought land use into the CExC was a more general evaluation of the cumulative exergy consumption of a process/system.

In general, the advanced CFB system (S1) was found to be more resource-efficient than the conventional CFB and MG system (S2 and S3). This can be explained from the perspective of the energy/materials recovered. If the MBT system and the EHE were used in the MSW CFB incineration plant, the output-useful resources increased, including ferrous and non-ferrous metals, the substitution of bricks, the produced electricity, and even the substitution of a soil conditioner or phosphate fertilizer for semi-compost. Therefore, the sum of the output, CExC, was higher than the conventional MSW incineration systems which could only achieve the recovery of electricity with a lower efficiency. Based on the results of the ELCA, the output, CExC, of the advanced CFB (S1) reached a value of 5365 MJ per t-MSW, while the value of S2 and S3 appeared to show lower values of 4338 MJ and 4593 MJ, respectively. On the contrary, the conventional CFB system (S2) had the lowest η_CExC_, which was due to the highest consumption of coal and diesel used for keeping the stable burning (1410 MJ of coal and diesel for S2, compared to less than 50 MJ for S1, and S3).

S1_soil conditioner_ appeared to be the best η_CExC_ among the advanced CFB scenarios, regardless of whether the NPP of land resources was considered or not. There were two main reasons for this result, one was that the S1_soil conditioner_ generated less fly ash than S1, leading to a lower consumption of the chelating agent. Additionally, the input, CExC, of the chelating agent decreased dramatically from 468 MJ of S1 to 343 MJ for S1_soil conditioner_. The other result was that the S1_soil conditioner_ recycled the semi-compost which could increase the CExC of 364 MJ, leading to the total CExC of the useful outputs reaching a higher value of 5529 MJ for S1_soil conditioner_, which was 5366 MJ for S1. Meanwhile, the input, CExC, reached the lower value of 8181 MJ for S1_soil conditioner_ and 8306 MJ for S1. As a result, the calculated η_CExC_ of S1_soil conditioner_ was higher than S1, while among the advanced CFB systerm, S1_landfill_ performed the worst due to the disposal of useful semi-compost. To be more specific, the output, CExC, of S1_landfill_ reached the value of 5165 MJ which was much lower than both S1 and S1_soil conditioner_.

With regard to the abatement exergy and AbatCExC efficiency (η_AbatCExC_) of different scenarios, the results showed that 58%, 61%, 56%, 37%, and 52%, of the AbatCExC efficiency were achieved by S1, S1_soil conditioner_ and S1_landfill_, S2, and S3, respectively. When taking land use into consideration, the values of the AbatCExC efficiency of S1, S1_soil conditioner_ and S1_landfill_, S2, and S3 reached 60%, 63%, 58%, 38%, and 54%, respectively. Similar to the difference calculated from the CExC efficiency, the produced electricity played an import role in calculation, which again revealed that the framework of the ELCA should take land use into consideration. The values of the AbatCExC efficiency were lower than the relevant CExC efficiency, due to the subtraction of the abatement exergy from the total output CExC. It was obvious that S2 consumed large amounts of coal and diesel and emitted the highest CO_2_ pollutants into the atmosphere as compared to S3 and S1, so the abatement exergy loss of S2 exhibited the highest value at 1200 MJ, while the abatement exergy of other systems (S1, and S3) was no more than 550 MJ. Additionally, the CExC efficiency and AbatCExC efficiency of S2 were the worst. With respect to the advanced CFB system (S1) and MG system (S3), S1 showed a better performance of AbatCExC efficiency than the conventional MG system, which could also be explained from the perspective of the energy/materials balance. Though the results of the AbatCExC efficiency seemed reasonable, the abatement exergy for the other emissions, especially heavy metals and dioxins, was lacking and the values were unavailable to obtain. Therefore, only the abatement exergy of CO_2_, SO_2_ and NO_x_ was considered in this study. However, the model and results could be easily updated if the data became available in the near future.

A parallel comparison about AbatCExC efficiency for the three advanced CFB scenarios (S1, S1_soil conditioner_ and S1_landfill_) was also conducted. It appeared that S1 led to an inferior performance than S1_soil conditioner_, and S1_landfill_ exhibited the worst result. As mentioned, S1_soil conditioner_ recycled semi-compost as a soil conditioner substituting phosphate fertilizer, which brought more gains of the output, CExC, than the CExC of the electricity recovered by semi-compost combusting in S1. Moreover, when taking abatement exergy into account, the consumed AbatCExC of S1_soil conditioner_ was lower than that of S1 due to less CO_2_, SO_2_ and NO_x_ emissions, indicating the material recovery as using a soil conditioner was a good method for the efficient utilization of semi-compost sorted from the MBT system. This was similar to the result and analysis of the CExC efficiency that S1_landfill_ performed the worst once more in the AbatCExC efficiency compared to S1 and S1_soil conditioner_.

The details, regardless of whether land use is considered, of the AbatCExC efficiency for different scenarios were calculated and presented in Supplementary Table S5 and the value of the AbatCExC efficiency decreased comparative to the relevant CExC efficiency. However, the changing tendency was similar.

**Figure 7:**
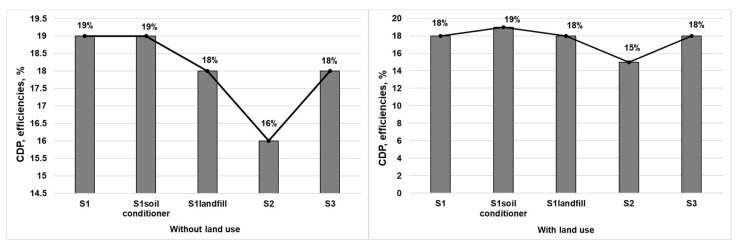
Cumulative degree of perfection (CDP) of the selected systems.

The CDP of the considered scenarios reflected that the consumed energy/materials derived from natural resources were recovered by the final useful products (mainly electricity). If land resources were seen as natural resources and the NPP of land use was embodied in the ELCA, the calculated CDP would be declined comparative to CDP from the conventional ELCA framework. Though the changing tendency may not change too much, the method considering land use was indeed a more scientific and holistic framework, which was efficient in analyzing all kinds of energy or material production processes, especially those with much land use, such as landfill site and industrial parks. From the definition, it was observed that CDP declined comparative to the CExC efficiency, which was the ratio of the total amount of CExC of the output and input flows. The CDP of the five selected scenarios, as shown in Figure 7, presented similar changing tendencies to the observation of the CExC efficiency. The value of CDP reached 19%, 19%, 18%, 16%, and 18% for S1, S1_soil conditioner_, S1_landfill_, S2 and S3, respectively, while the values were slightly lower when land use was considered. The results and analysis of the CDP ranking were similar to the CExC efficiency ranking, where the advanced CFB system (S1) was superior to the conventional CFB (S2), and the MG (S3) system and conventional CFB (S2) system had the lowest efficiency.

As the CDP indicates the thermodynamics perfection and the degree of the renewability of a system or a process, as well as quantifying the energy utilization efficiency at a life cycle level [21], the advanced CFB system (S1) showed a higher thermodynamics perfection and degree of renewability. S2 showed the lowest thermodynamics perfection and degree of renewability because of the large amounts of coal and diesel that were used. Once again, S1_soil conditioner_ performed the best and had the highest CDP among advanced CFB scenarios due to the recycled semi-compost as a soil conditioner substituting phosphate fertilizer and other useful resources, as well as a high recovery of electricity.

## 4. Conclusions

For the sake of applying the life cycle assessment to an advanced circulating fluidized bed incineration system, five scenarios were proposed and parallel comparisons were conducted from the environmental impacts and exergetic efficiency perspectives using the methods of LCA and ELCA.

The results showed that the advanced circulating fluidized bed incineration system (S1) exhibited higher net environmental savings and CExC efficiencies than conventional circulating fluidized beds (S2) and moving grate (S3) incineration systems due to a higher energy and material recovery.

The different energy and material recovery waste treatment scenarios in the process improvements were assessed. The scenario (S1_soil conditioner_) using semi-compost as a soil conditioner obtained the highest net environmental savings, CExC efficiency, CDP and AbatCExC efficiency, indicating the positive improvements which could be achieved if semi-compost was used for material recovery rather than energy recovery.

The ELCA was also conducted based on the consideration of land use. It was observed that a more thorough and scientific evaluation could be achieved with the combination of land resources and ECLA.

## Figures and Tables

**Table 1:** Characteristics of MSW.

	MSW Characteristics (wt.%, as Received Basis)							
	Carbon	Hydrogen	Nitrogen	Sulfur	Chloride	Moisture	Ash	LHV ^a^ (MJ/kg)
Chinese average level	16.69	2.3	0.45	0.11	0.37	48.12	22.6	5.337
S1: advanced CFB	17.3	2.59	0.3	0.2	0.45	49.4	18.94	5.851
S2: CFB incineration	16.4	2.28	0.25	0.13	0.44	48.73	22.6	5.224
S3: MG incineration	16.9	2.43	0.3	0.13	0.42	50.97	20.44	5.67

^a^ LHV: lower heating value.

## Data Availability

The data presented in this study for calculation are available in this manuscript and Supplementary Materials. While the raw data are available on request from the corresponding author, and are not publicly available due to enterprise privacy.

## Supplementary Materials

The following are available online at www.mdpi.com/article/10.3390/ijerph181910432/s1, Figure S1: The technological process of advanced CFB incineration system, Table S1: Semi-compost Characteristics Employed in S1, S1soil conditioner and S1landfill scenario; Figure S2: The technological process of MG incineration system (S3), Table S2: Limited values for heavy metals in soil conditioner GB/T 23349-200; Figure S3: The figuration of EHE: 1—ash inlet 2—ash back 3—equalizing air chamber 4—high temperature superheater outlet header 5—high temperature superheater inlet header 6—high temperature superheater serpentine tube 7—ash back overflow hole, Table S3: Inventory of each scenario for the treatment of 1 t-MSW; Table S4: Cumulative exergy consumption (CExC) and abatement exergy (AbatEx) of materials/energy flows and air emissions, as well as the net primary production (NPP) of land use; Table S5: The total abatement exergy loss of the considered scenarios and relevant AbatCExC efficiency with or without considering land use.
