# Supplementary material for: Life Cycle Assessment of Advanced Circulating Fluidized Bed Municipal Solid Waste Incineration System from an Environmental and Exergetic Perspective
Source: Int J Environ Res Public Health. 2021 Oct 3;18(19):10432. doi: 10.3390/ijerph181910432 (PMC8508379; doi:10.3390/ijerph181910432)
Supplement: Supplementary file 1 [file ijerph-18-10432-s001.zip › ijerph-1333329-supplementary.pdf]

Life Cycle Assessment of Advanced Circulating Fluidized Bed Municipal Solid Waste Incineration System from an Environmental and Exergetic Perspective

Jun Li [1], Lixian Wang [1], Yong Chi [1,*], Zhaozhi Zhou [2], Yuanjun Tang [3] and Hui Zhang [1]

Supplementary Material

Data description

The dataset in this supplementary material describes the characteristics of semi-compost sorted from MBT system used in S1, $S1_{soil\ conditioner}$ and $S1_{landfill}$ scenario (Table S1); the Chinese National Regulations of limited values for heavy metals in soil conditioner GB/T 23349-200 (Table S2); the life cycle inventories of the five scenarios from plant daily operating records(Table S3); the background data of exergetic life cycle assessment in this study (Table S4); the total abatement exergy loss of the considered scenarios and relevant AbatCExC efficiency with or without considering land use (Table S5); the technological process of advanced CFB incineration system (Fig. S1); and, the technological process of MG incineration system (Fig. S2); the figuration of external heat transfer (Fig. S3).

Table S1

Semi-compost Characteristics Employed in S1, $S1_{soil\ conditioner}$ and $S1_{landfill}$ scenario

Parameter	value	Unit
moisture content	27.46	%
Ash content	52.33	%
C	8.32	%
H	0.57	%
N	0.59	%
S	0.17	%
O	10.65	%
K	0.59	%
P	0.16	%
Cr	7.37E-03	%
Cu	5.33E-03	%
Mn	1.35E-02	%
Ni	9.07E-04	%
Pb	3.04E-03	%
Zn	2.19E-02	%
Lower heating value	1.62	MJ/kg

Table S2

Limited values for heavy metals in soil conditioner GB/T 23349-200	
As (%)	0.005

Cd (%)	0.001
Pb (%)	0.02
Cr (%)	0.05
Hg (%)	0.0005

Table S3

Inventory of each scenario for the treatment of 1 t-MSW

Parameter	Unit	Scenarios				
		S1	$S1_{soil\ conditioner}$	$S1_{landfill}$	S2	S3
Input						
Electricity	MJ	343.01	343.01	343.01	323.28	273.71
Diesel for mechanical operation	kg	0.53	0.53	0.53	2.06	0.25
Diesel for incineration startup	kg	1.50E-01	1.50E-01	1.50E-01	1.49E-01	1.49E-01
Coal	kg				41.80	
Hydrated lime	kg	2.34	2.34	2.34	11.45	15.13
Activated carbon	kg	6.00E-01	6.00E-01	6.00E-01	2.65E-01	5.70E-01
Titanium dioxide	kg				5.08E-02	4.72E-01

Ammonia water (20%)	kg	1.49	1.49	1.49	1.102	4.27
Chelating agent	kg	2.35	1.72	1.72	0.403	0.66
Cement	kg				1.86	5
Lubricating oil	kg	2.84E-03	2.84E-03	2.84E-03	4.00E-02	1.69E-03
Tap water	kg	3.79	3.79	3.79	20.73	3.79
High density polyethylene	kg			1.55E-02		
Clay	kg			3.74		
Output						
Electricity	MJ	1372.07	1336.86	1336.86	1252.80	1198.04
Ferrous metal	kg	4.13	4.13	4.13		
Non-ferrous metal	kg	1.59	1.59	1.59		
Soil conditioner	kg		83.65			
Bricks	kg	89.06	65.20	65.20	81.67	200.00
PM10[a]	kg	1.04E-02	9.53E-03	9.53E-03	4.10E-02	9.16E-03
Carbon dioxide, fossil	kg	2.37	2.37	2.37	111.75	1.32
Sulfur dioxide	kg	3.02E-02	3.02E-02	3.02E-02	4.50E-02	1.04E-01
Nitrous oxide	kg	3.86E-01	3.70E-01	3.70E-01	3.26E-01	3.69E-01
Hydrogen chloride	kg	5.86E-02	5.86E-02	5.86E-02	1.58E-02	4.78E-02
Hydrogen fluoride	kg	1.92E-03	1.92E-03	1.92E-03	8.91E-03	4.96E-02

Carbon monoxide	kg	1.28E-01	1.28E-01	1.28E-01	2.00E-01	1.82E-01
Mercury	kg	7.20E-08	7.20E-08	7.20E-08	2.17E-06	1.78E-08
Cadmium	kg	2.36E-06	1.80E-06	1.80E-06	5.55E-06	4.00E-06
Arsenic	kg	5.18E-06	3.97E-06	3.97E-06	1.46E-05	4.31E-06
Lead	kg	1.62E-05	1.23E-05	1.23E-05	5.55E-06	8.88E-06
PCDD/DFs[a]	kg TEQ	2.88E-11	2.88E-11	2.88E-11	1.32E-10	2.35E-10
Ammonia	kg	1.33E-02	1.33E-02	6.13E-01	2.44E-02	1.11E-02
Hydrogen sulfide	kg	4.55E-03	4.55E-03	4.55E-03	2.77E-04	1.56E-04
Methane	kg			2.96		
COD[a]	kg	4.90E-02	4.89E-02	4.91E-02	4.35E-02	4.32E-03
NH3-N[a]	kg	4.90E-03	4.89E-03	4.91E-03	3.23E-03	4.17E-05
BOD[a]	kg	9.80E-03	9.78E-03	9.81E-03	4.42E-03	1.88E-04
Fly ash	kg	73.90	54.10	54.10	83.30	60.00
Non-combustible residues	kg	122.33	122.33	122.33		22.30

[a] PM_{10}: particulate matter of diameter less than 10 micrometer; PCDDs: polychlorinated dibenzo-p-dioxins; PCDFs: polychlorinated dibenzo-dioxins; COD: chemical oxygen demand; NH_3-N: ammonia nitrogen; BOD: biochemical oxygen demand. The data of S1, $S1_{soil\ conditioner}$, S2, S3 were collected in real operated situation. The data of $S1_{landfill}$ comes from work of Sundqvist[1]. This was cleared under table S3.

Table S4

Cumulative exergy consumption (CExC) and abatement exergy (AbatEx) of materials/energy flows and air emissions, as well as the net primary production (NPP) of land use.

Parameter	CExC	NPP of land resource	Chemical exergy	Abatement exergy	unit	Reference
Electricity	11.61	0.46	3.6		MJ/kWh	[2]
Coal	30.44	0.118			MJ/kg	[3]
Diesel	67.2	6.93			MJ/kg	[2]
MSW			β×LHV[a]		MJ	
Hydrated lime	9.96	0.236			MJ/kg	[3]
Activated carbon	247	3.9			MJ/kg	[4]
Titanium dioxide	323.47	4.48			MJ/kg	GaBi 8.0
Ammonia water (20%)	12.56	0.06			MJ/kg	GaBi 8.0
Chelating agent	199.5	2.92			MJ/kg	GaBi 8.0
Cement	6.18	0.57			MJ/kg	[3]
Lubricating oil	80.11	0.214			MJ/kg	GaBi 8.0
Tap water	80.11	0.262			MJ/m3	GaBi 8.0
High density polyethylene	97.71	0.277			MJ/kg	GaBi 8.0

Clay		0	0.69		MJ/kg	[5]
Ferrous metal	54	0.5	7		MJ/kg	[6]
Non-ferrous metal	246.78	0.82	32.93		MJ/kg	[7]
Bricks	3.65	0.02	0.952		MJ/kg	[8]
Soil conditioner	4.36	0.086	1.157		MJ/kg	[8]
CO_2[a]				5.86	MJ/kg	[9]
SO_2[a]				57	MJ/kg	[9]
NO_x[a]				16	MJ/kg	[9]

[a] CExC of MSW was not considered, since the LCA and ELCA studies started from MSW entering WtE plants in this work, therefor only chemical exergy was considered; LHV: lower heating value.

[a] Due to the lack of available data, only the abatement exergy of the gases emissions CO_2, SO_2 and NO_x was considered.

[a] The chemical exergy of MSW can be calculated by Eq. (1) and (2) [10].

$$e = \beta \times LHV_{MSW} \tag{1}$$

$$\beta = \frac{1.0412 + 0.216H/C - 0.2499O/C \times (1 + 0.7884H/C) + 0.045N/C}{1 - 0.3034O/C} \quad (O/C \le 2.67) \tag{2}$$

Table S5

The total abatement exergy loss of the considered scenarios and relevant AbatCExC efficiency with or without considering land use.

Scenarios	Emissions	Amount (kg)	Abatement exergy	AbatCExC efficiency	
				considering NNP of land use	not considering NNP of land use
S1	CO2	89.4	547.779	59.8%	58.0%
	SO2	0.255			
	NOx	0.585			
$S1_{soil\ conditioner}$	CO2	87.6	536.724	62.9%	61.0%
	SO2	0.252			
	NOx	0.564			
$S1_{landfill}$	CO2	88.9	544.682	56.5%	58.2%
	SO2	0.256			
	NOx	0.571			
S2	CO2	201	1200.95	36.7%	38.3%
	SO2	0.258			
	NOx	0.524			
S3	CO2	85.9	527.969	52.0%	53.8%
	SO2	0.283			
	NOx	0.529			

Specific descriptions of the five scenarios

S1: Advanced circulating fluidized bed incineration system in Zibo

S1 is the advanced type of circulating fluidized beds in China, supported by complicated mechanical biological treatment system for treating raw waste before combustion, was designed to reach a relative high steam parameters level (520 ℃, 79 bar). Mechanical biological treatment (MBT) of mixed streams is becoming increasingly popular as a method for treating MSW [11]. The outputs of MBT plants are: recyclable (mostly metals) and compostable materials, Refuse Derived Fuel (RDF) and a fraction of residuals. Differently, the selected plant S1 took MBT as a part to treat raw waste, the advantage was that after biological reaction and mechanical screening RDF with low water content and high calorific value could be obtained, also useful resources like ferrous and non-ferrous (aluminum) metals, as well as semi-compost burnt in incinerator. The MSW underwent initial screening, for the separation of ferrous metals, then was sent to drying house for biological fermentation drying which usually lasts for 7 days. According to on-site operational records, the waste input to drying house had water content at 50~70%, after biological drying it had less water content usually at 30% and higher calorific value at 10 MJ / kg compared to 5.8 MJ / kg-raw waste. The ferrous and non-ferrous metals as well as semi-compost sorted from MBT were recovered, and were seen as gains because they can offset the environmental impacts caused by relevant production processes. Other heavy residues like brick fractions are sent to landfill site. The reason why S1 can reach a relatively high steam parameters is that the final steam superheater takes place in the heat exchangers

located in the solids returns as shown in Fig. S1, so prevents the contact between high temperature flue gas and heat transfer, as a result, the configuration not only guarantees high temperature and pressure steam but also reduces fouling and corrosion of the heat transfer surfaces. The advanced CFB incineration system (S1) dealt with 536, 000 tons of raw waste annually, and had a lifetime of 30 years. It produced power electricity 381 kWh per t-MSW and 24.9% of the generated electricity was consumed in the plant, which is relative higher than usual value 20% [12], because the MBT part is energy-intensive. The produced RDF was 492 kg per t-MSW, thus the power generation efficiency was approximately at 28%. As for the air pollution control system, S1 was composed of Selective Non-Catalytic Reduction technology and semidry-dry scrubber, active carbon absorption as well as bag house filter. The furnace slags were 81 kg per t-MSW seen as a kind of resource sold for money, and the fly ash were fixed by chelating agents and then sent together with non-combustible residues to landfill site.

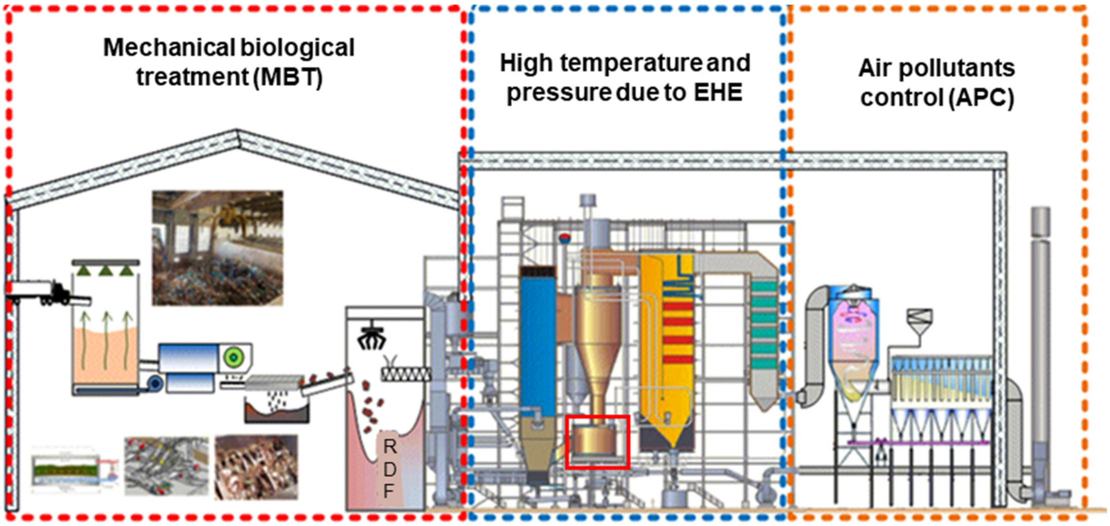

Figure S1. The technological process of advanced CFB incineration system

$S1_{soil\ conditioner}$: Advanced circulating fluidized bed incineration system with semi-compost

used as soil conditioner in Zibo

Generally, one potential advantage of MBT technology over waste incineration plant was the production of semi-compost that could be used as P fertilizer or soil conditioner which are good for soil and planting. Thus, $S1_{soil\ conditioner}$ with the utilization of semi-compost sorted from MBT as soil conditioner for farmland was also proposed and evaluated in this study, even though its commercial application is still under research. The incineration process of $S1_{soil\ conditioner}$ was the same as S1, only different were the input fuels, S1 was 492 kg RDF and 83.6 kg semi-compost per t-MSW, while $S1_{soil\ conditioner}$ was 492 kg RDF per t-MSW. According to S1, the power generation efficiency was 28%. The $S1_{soil\ conditioner}$ power generation efficiency was similar and assumed as 28%, thus the generated electricity was 371 kWh per t-MSW and internal electricity consumption remained unchanged as 95 kWh per t-MSW. Obviously, it is known that auxiliary energy was the same in $S1_{soil\ conditioner}$ as compared to S1, since the MBT system worked under the same circumstance. However, due to more fuels put into incinerator, S1 generated more electricity than $S1_{soil\ conditioner}$, and $S1_{soil\ conditioner}$ obtained resource utilization of 83.65 kg semi-compost per t-MSW. APC system and the treatment of solid residues of $S1_{soil\ conditioner}$ were almost the same as S1.

$S1_{landfill}$: Advanced circulating fluidized bed incineration system with landfill disposal of semi-compost in Zibo

$S1_{landfill}$ represented to the advanced CFB system which sent the produced semi-compost to landfill site in Zibo. The landfill disposal of semi-compost was chosen because on one hand, semi-compost sorted from MBT system had high content of ash which could cause adverse

effects to the combustion; on the other hand, semi-compost could be used for fertilizer/soil conditioner or not was under studying. Similar to $S1_{soil\ conditioner}$, the total generated electricity and internal power consumption were 371kWh and 95 kWh per t-MSW, respectively. However, the landfill disposal of residues was higher in $S1_{landfill}$ compared to $S1_{soil\ conditioner}$, since the semi-compost were sent to landfill site. Compared to S1 and $S1_{soil\ conditioner}$, the same APC system and solid residues treatment methods were adopted in $S1_{landfill}$.

S2: Conventional circulating fluidized bed incineration system in Hangzhou

S2 represents the conventional circulating fluidized bed MSW incineration system in Hangzhou, China, with parameters of the boiler (485 ℃, 53 bar), and the production of electricity (24 MW), whose commercial operation was started in 2015, and approximately 400,000 tons of MSW can be handled annually. Raw MSW was first collected and transported to the plant depositories for pre-treatment, including drying, shredding, and/or palletisation, then was sent to furnace for combustion. High calorific fuels such as coal and diesel were needed to start the incineration, the consumption of auxiliary fuels are 41.8 kg coal and 2.2 kg diesel per t-MSW. The processed waste had intensive contact with the bed material in the chamber at 850~1000 ℃, and the produced flue gas was kept in the chamber for at least for 2 seconds for preventing dioxins formation. Though S2 also had an external heat exchanger to get superheat steam, but the produced steam were much lower than S1 (485 ℃, 53 bar for S2 comparing to 520 ℃, 79 bar for S1), and S2 had no pretreatment for raw waste, so in this study S2 were regarded as conventional CFB system. In order to control the emission of flue gas pollutants, the selective non-catalytic reduction equipment and selective catalytic reduction

system were installed, as well flue gas treatment facilities composed of semidry scrubber, active carbon absorption, and bag house filters were used [13]. The emissions at stack met the national regulation. For solid residues disposal, the produced bottom slags were estimated to be 89 kg per t-MSW and sold as resources, and stabilized ash from the mixing of fly ash and cement or chelating agent were landfilled. The overall power generation is 348 kWh per t-MSW and the total consumption of electricity is 90 kWh per t-MSW, accounting for 25.8% of the total generated electricity, which is higher than the usual ratio of 20% [14].

S3: Moving grate incineration system in Zhuji

S3 reflects the MG incineration system in Zhuji, China, which was built to substitute old CFB incineration plant for improvement of technology and environment protection. As shown in Fig. S2, the plant based on mechanical moving grate (MG) for the production of electricity (24MW), and S3 is just a half of completion of the whole construction. The selected MG system, with an annual capacity of 147, 782 t, was assumed to be in operation for 30 y as designed. Incinerators in the world are predominately MG type. A main advantage of MG incinerator is its capacity to the treat unsorted waste [15]. Raw waste collected from urban district was pushed over the grate, experiencing consecutively drying, devolatilization, and combustion along the moving grate. S3 had an ingenious architecture of chamber, which can accelerate the mix between flue gas, guarantee the complete combustion of flue gas, and can keep the flue gas staying in the chamber for at least 2 seconds for preventing dioxins formation. Waste leachate was divided into two parts after a series of treating process, one was clear water and was collected to the waste water treatment plant, the other was concentrated water which could be

used for the stabilization of fly ash or injected into chamber for combustion. Heat recovery steam generates superheated steam (450 ℃, 53 bar) with the utilization of heat from flue gas, and steam pushes turbine generator to produce electricity. Energy requirement of the plant was assumed to be supplied internally. According to the operational records, electricity produced was approximately 333 kWh per t-MSW, and internal electricity consumption rate and quantity reaches 22.8% and 76 kWh per t-MSW, respectively. Regarding to air pollution control (APC) systems, S3 equipped with semi-dry flue gas treatment system (SNCR-SCR, semidry-dry scrubber, active carbon absorption, and bag house filter). Furnace slags were 200 kg per t-MSW and collected for recovery, and ash from bag house filters was fixed by chelating agents and then sent to landfill site.

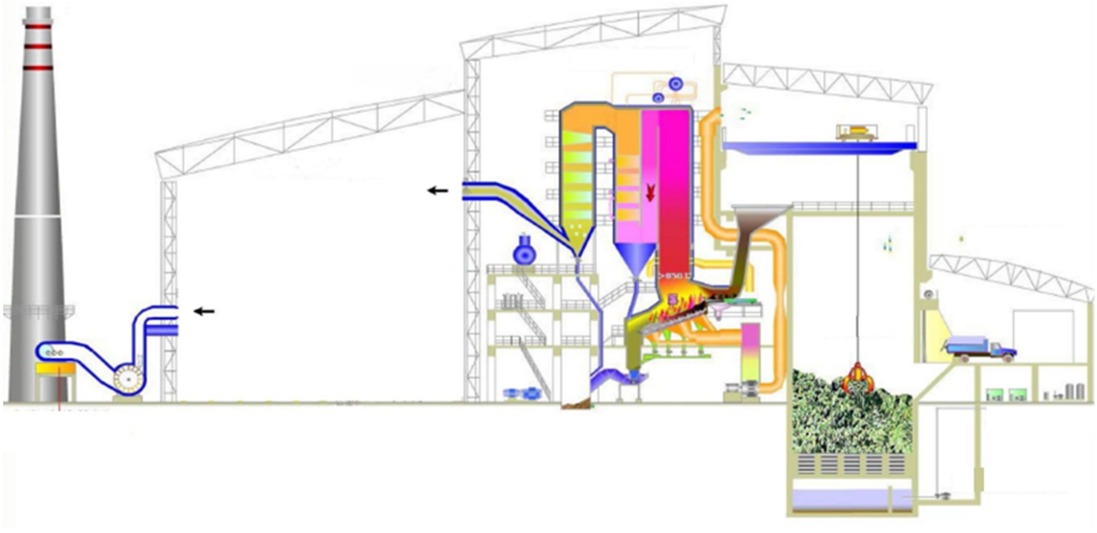

Figure S2. The technological process of MG incineration system (S3)

Detailed description concerning MBT and EHE

For the part of MBT system, Mechanical biological treatment (MBT) of mixed streams is becoming increasingly popular as a method for treating MSW [11]. The outputs of MBT plants are: recyclable (mostly metals) and compostable materials, Refuse Derived Fuel (RDF) and a fraction of residuals. The biological drying of waste is a process of drying pre-crushed mixed garbage using the principle of composting. In the case of forced ventilation, microorganisms use the perishable organic matter in the mixed waste to ferment and produce heat energy. Ventilation at high temperatures accelerates the volatilization of water leading to the decreasing of water contain in waste. Differently, the selected plant S1 took MBT as a part to treat raw waste, the advantage was that after biological reaction and mechanical screening RDF with low water content and high calorific value could be obtained, together with useful resources like ferrous and non-ferrous (aluminum) metals, as well as semi-compost burnt in incinerator.

For the part of EHE system, as shown in Fig. S3, the ash particle with high temperature enters EHE through ash inlet (1). In the EHE, there are serpentine heat exchanger tubes (6) which are used to heat steam. Low temperature steam gets in through inlet header (5) and after heated by ash, steam with higher temperature was gets through outlet header (4). The air chamber (3) can make ash flow in EHE and the ash can get out through ash back overflow hole (7) and finally circulates through ash back outlet (2).

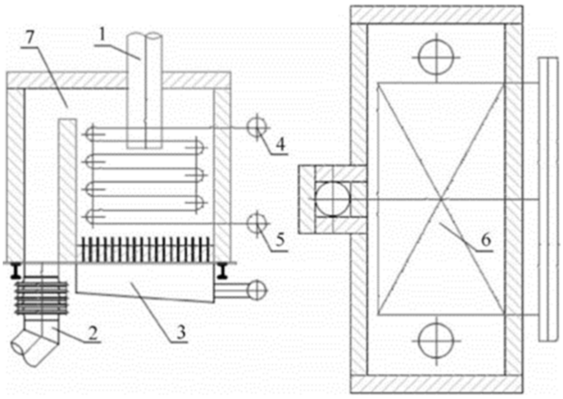

Figure S3. The figuration of EHE: 1- ash inlet 2 – ash back 3- equalizing air chamber 4- high temperature superheater outlet header 5- high temperature superheater inlet header 6- high temperature superheater serpentine tube 7- ash back overflow hole.

References

[1] Sundqvist, J. O., Finnveden, G., Stripple, H., Albertsson, A. C., Karlsson, S., Berendson, J., & Hoeglund, L. O. (1997). Life cycle assessment and solid waste: stage 2: annual report. AFR-Report (Sweden). https://agris.fao.org/agris-search/search.do?recordID=SE1998010344

[2] Sun B, Nie Z, Gao F. et al. (2014) Cumulative exergy consumption (CExC) analysis of energy carriers in China[J]. International Journal of Exergy,15(2):196-213. https://doi.org/10.1504/IJEX.2014.06564.

[3] Szargut, J.; Morris, D. R.; Steward, F. R. (1987) Cumulative exergy consumption and cumulative degree of perfection of chemical processes. International Journal of energy research. https://doi.org/10.1002/er.4440110207.

[4] Elvers B, Hawkins S, Arpe H. Ullman's encyclopedia of indus-trial chemistry. VCH Verlagsgesellschaft, 1996.

[5] Sun, B., Liu, Y., Nie, Z. et al. (2020) Exergy-based resource consumption analysis of cement

clinker production using natural mineral and using calcium carbide sludge (CCS) as raw material in China. Int J Life Cycle Assess 25, 667–677 (2020). https://doi.org/10.1007/s11367-019-01725-4.

[6] Dewulf, J., Van Langenhove, H., & Dirckx, J. (2001). Exergy analysis in the assessment of the sustainability of waste gas treatment systems. Science of the Total Environment, 273(1–3), 41–52. https://doi.org/10.1016/S0048-9697(00)00841-X.

[7] Sun, B., Nie, Z., Gao, F. et al. (2015) Cumulative exergy demand analysis of the primary aluminum produced in China and its natural resource-saving potential in transportation. Int J Life Cycle Assess 20, 1048–1060 (2015). https://doi.org/10.1007/s11367-014-0828-9.

[8] Szargut, J., 2005. Exergy Method – Technical and Ecological Applications. WIT Press, Southhampton, UK.Zhou, Z., Chi, Y., Dong, J., Tang, Y., & Ni, M. (2019). Model development of sustainability assessment from a life cycle perspective: A case study on waste management systems in China. Journal of Cleaner Production, 210, 1005–1014. https://doi.org/10.1016/j.jclepro.2018.11.074.

[9] Cornelissen RL. Thermodynamics and sustainable development: the use of exergy analysis and the reduction of irreversibility. Universiteit Twente; 1997. http://purl.utwente.nl/publications/32030.

[10] Prins, M. J., Ptasinski, K. J., & Janssen, F. J. J. G. (2003). Thermodynamics of gas-char reactions: First and second law analysis. Chemical Engineering Science, 58(3–6), 1003–1011. https://doi.org/10.1016/S0009-2509(02)00641-3.

[11] Farrell, M., & Jones, D. L. (2009). Critical evaluation of municipal solid waste composting and potential compost markets. In Bioresource Technology (Vol. 100, Issue 19, pp. 4301–4310).

Elsevier. https://doi.org/10.1016/j.biortech.2009.04.029.

[12] Morris, M., & Waldheim, L. (1998). Energy recovery from solid waste fuels using advanced gasification technology. Waste Management, 18(6–8), 557–564. https://doi.org/10.1016/S0956-053X(98)00146-9.

[13] Zhou, Z., Chi, Y., Dong, J., Tang, Y., & Ni, M. (2019). Model development of sustainability assessment from a life cycle perspective: A case study on waste management systems in China. Journal of Cleaner Production, 210, 1005–1014. https://doi.org/10.1016/j.jclepro.2018.11.074.

[14] Dong, J., Tang, Y., Nzihou, A., Chi, Y., Weiss-Hortala, E., & Ni, M. (2018). Life cycle assessment of pyrolysis, gasification and incineration waste-to-energy technologies: Theoretical analysis and case study of commercial plants. Science of the Total Environment, 626, 744–753. https://doi.org/10.1016/j.scitotenv.2018.01.151.

[15] Lu, J. W., Zhang, S., Hai, J., & Lei, M. (2017). Status and perspectives of municipal solid waste incineration in China: A comparison with developed regions. In Waste Management (Vol. 69, pp. 170–186). Elsevier Ltd. https://doi.org/10.1016/j.wasman.2017.04.014.
